# Melatonin: a natural guardian in cancer treatment

**DOI:** 10.3389/fphar.2025.1617508

**Published:** 2025-07-18

**Authors:** Yurou Cao, Hang Zhang, Xubin Chen, Conghui Li, Jingxin Chen

**Affiliations:** ^1^ School of Stomatology, Hainan Medical University and Hainan Academy of Medical Sciences, Haikou, Hainan, China; ^2^ Department of Stomatology, Hainan General Hospital (Hainan Affiliated Hospital of Hainan Medical University), Haikou, China

**Keywords:** melatonin (MLT), anticancer activity, antioxidant activity, chemotherapy synergy, targeted delivery

## Abstract

Melatonin (MLT), a naturally occurring hormone produced by the pineal gland, exhibits significant anticancer effects. It has superior antioxidant, inhibit tumor cell proliferation, migration, angiogenesis-inhibiting, and tumor cell apoptosis-inducing functions. Mechanistically, melatonin inhibits tumor development through epigenetic regulation, metabolic reprogramming, immune micro-environment, and regulation of important signaling pathways (PI3K/AKT). In addition, MLT significantly enhances anticancer efficacy in combination with other anticancer drugs, such as cisplatin, 5-fluorouracil, and paclitaxel. However, the shortcomings of melatonin, such as its low bioavailability, rapid metabolism, and significant individual variation in secretion, have limited its clinical application in anticancer therapy. This limitation has been mitigated by targeted delivery and individualized therapy. Therefore, MLT may be a promising candidate for natural hormone therapy in the future.

## 1 Introduction

**FIGURE 1:**
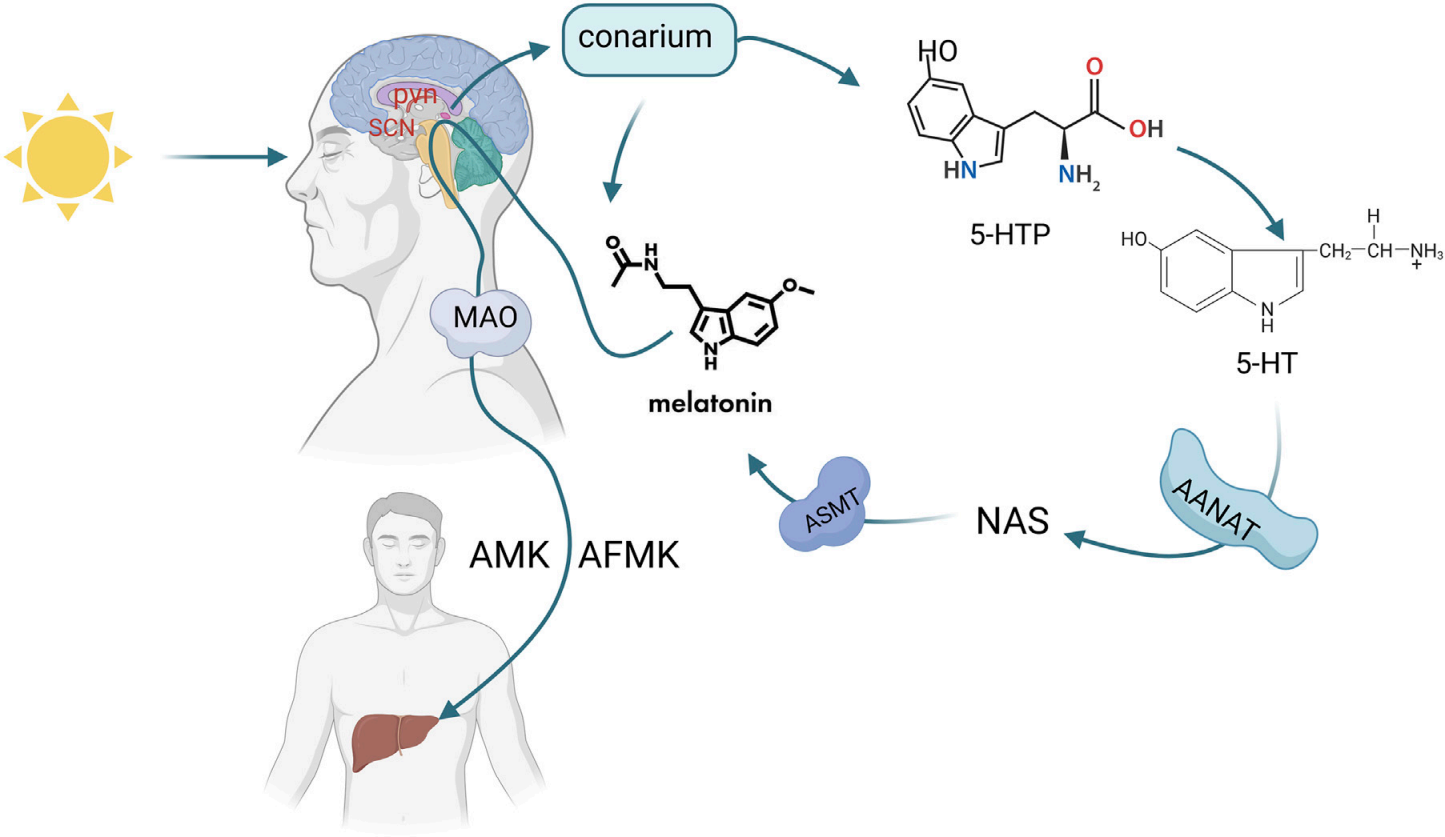
Synthesis and metabolic pathway of melatonin (Melatonin (MLT) synthesis is triggered by light via the retina-SCN-pineal pathway. Tryptophan converts to MLT through enzymatic steps, then enters blood and CSF, metabolizing into AMK/AFMK in the liver). Created in BioRender. cao, y. (2025) https://BioRender.com/p10x701.

Melatonin (MLT) is an indoleamine secreted by the pineal gland and other organs (retina, gastrointestinal tract, lymphocytes, etc.) (Ahmad et al., 2023). Melatonin secreted by the pineal gland is mainly regulated by light exposure, which activates a pathway starting from the retina, transmitting signals to the suprachiasmatic nucleus (SCN) in the hypothalamus, then to the paraventricular nucleus (PVN), brainstem, and spinal cord, and finally to the pineal gland (Vasey et al., 2021). Tryptophan is the precursor for MLT synthesis, entering the pineal gland through the bloodstream and being converted into 5-hydroxytryptophan (5-HTP) by tryptophan hydroxylase. 5-HTP is further converted into 5-hydroxytryptamine (5-HT) by aromatic L-amino acid decarboxylase (AADC), which is then converted to N-acetyl-5-hydroxytryptamine (NAS) by arylalkylamine N-acetyltransferase (AANT), and finally to MLT by acetylserotonin O-methyltransferase (ASMT). This MLT enters the cerebrospinal fluid and bloodstream (Figure 1).

Melatonin secreted by the pineal gland is related to the duration of darkness. The main function of melatonin is to transmit darkness signals, which may regulate circadian rhythms and seasonal changes (Claustrat et al., 2005). These circadian rhythms are regulated by the suprachiasmatic nucleus (SCN) of the hypothalamus. The light-dark cycle of the environment plays a key role in the synchronization of the SCN (Pandi-Perumal et al., 2008). MLT synthesis is affected by light, sleep, analgesic drugs, and other factors (Ahmad et al., 2023). Melatonin secretion peaks at night until 3:00 a.m. at the age of 1–3 years, and declines by 80% in adulthood. Seventy% of the melatonin secreted by the pineal gland is metabolized by the liver (Waldhauser et al., 1993). According to reports, in mammals, less than 5% of melatonin is produced by the pineal gland (Reiter et al., 2024). However, most melatonin is secreted outside the pineal gland (in the retina, skin, gastrointestinal tract, immune cells, mitochondria, etc.) and is not affected by circadian rhythms (Acuña-Castroviejo et al., 2014). Due to this characteristic, some researchers have speculated that the local production of melatonin outside the pineal gland plays a more direct and sustained role in the tumor micro-environment, while melatonin secreted by the pineal gland directly and indirectly regulates tumor development during nighttime secretion (Bonmati-Carrion and Tomas-Loba, 2021).

In mammals, melatonin (MLT) activates membrane-bound G protein-coupled receptor (GPCR) receptor binding (MT1, MT2) or by direct action (Jockers et al., 2016). MT1 is a major distribution site in the suprachiasmatic nucleus (SCN), hippocampus, and amygdala (Jockers et al., 2008), and the MTI secreted by the SCN is subject to circadian rhythms. MT2 has a restricted distribution and is mainly confined to the retina. Melatonin has a greater affinity for MTI than MT2 (Liu et al., 2016). It was found that the binding conformation of MTI with 2-iodohydroxytryptamine or ramelteon was more favorable for the binding of high-affinity ligands, and H5.42 and N4.56 of MT2 had weaker affinity due to sequence differences (Wang Q. et al., 2022). These receptors are directly or indirectly linked to a variety of different signaling pathways, thereby inhibiting the response of cancer cells.

## 2 Antioxidant activity

As a potent redox regulator, melatonin (MLT) exhibits dual antioxidant mechanisms: executing direct ROS/RNS neutralization while concurrently upregulating endogenous antioxidant enzymatic systems through catalytic proficiency modulation, and its main antioxidant effect is the formation of N-acetyl-5-methoxytryptophan (AMK), deformed from the metabolite of melatonin, N1-acetyl-N2-formyl-5-methoxykynurenine (AFMK) (Galano et al., 2013). Melatonin is most concentrated in cell membranes (Venegas et al., 2012),is highly concentrated in mitochondria, and protects proteins, lipids, and DNA from free radical-induced oxidative damage, in addition to preventing mutations and damage to mitochondrial DNA (García et al., 2014).

Mitochondria are the primary sites of ROS production. Within mitochondria, melatonin exerts its antioxidant function by directly scavenging free radicals and also influences the mitochondrial membrane potential to prevent damage from oxidative stress (Chitimus et al., 2020). Melatonin protects the electron transport chain (ETC) by binding to the Fe-S cluster of NADPH dehydrogenase, reducing the generation of superoxide anion radicals (O2•−) caused by electron leakage, blocking the opening of the mitochondrial permeability transition pore (mPTP), and preventing apoptosis caused by cytochrome C leakage (Hardeland, 2017; Tan et al., 2007). Melatonin orchestrates tumor-selective reverse electron transport through mitochondrial Complex I in head and neck squamous cell carcinoma (HNSCC), eliciting site-specific bioenergetic disruption via modulation of NADH/ubiquinone oxidoreductase flux, thereby augmenting ROS-mediated activation of the intrinsic apoptotic cascade through redox-sensitive BAX oligomerization and cytochrome c efflux (Florido et al., 2022a). In addition, the deacetylase sirtuin 3 (Sirt3) increases the content of the pyruvate dehydrogenase complex (PDH) through deacetylation, thereby participating in ATP production. PDH significantly enhances mitochondrial energy metabolism (Ozden et al., 2014). Melatonin enhances superoxide dismutase 2 (SOD2) activity through SIRT3-mediated deacetylation, accelerating the conversion of O2•− to H2O2 (Ning et al., 2022). MLT reverses the Warburg effect and inhibits lung cancer progression in lung cancer cells by stimulating Sirt3 to increase PDH production (Chen X. et al., 2021).

Melatonin exerts its anticancer effects through a multi-level antioxidant mechanism. ROS triggers apoptosis by activating the pro-apoptotic proteins caspase-3/7/9 and cleaved PARP, disrupting mitochondrial function. In pancreatic cancer, melatonin-induced ROS enhances apoptosis through the mitochondrial pathway. ROS inhibits cancer cell invasion and migration by regulating the key JAK2/STAT3 signaling pathway. Additionally, cancer cells combat ROS by relying on their antioxidant system. In hepatocellular carcinoma cells, melatonin increases ROS accumulation and promotes apoptosis by inhibiting GSH levels (Ordoñez et al., 2015). In hepatocellular carcinoma cells, MLT induces hepatocellular carcinoma cell death by increasing ROS production. Concomitant use of melatonin with cisplatin promotes ROS generation and increases cervical cancer cell death (Pariente et al., 2016). Melatonin enhances ROS production in botulinic acid-induced oral squamous cell carcinoma (OSCC) with concomitant activation of DNA repair (Shih et al., 2021).

In addition, melatonin is conditioned to be pro-oxidant. High concentrations of melatonin promote ROS generation. Studies have shown that melatonin promotes ROS production depending on cell type, concentration and duration of action. High concentrations of melatonin are pro-oxidant in cancer cells, but do not increase ROS production in lymphocytes (Büyükavci et al., 2006). The longer a high concentration of melatonin acts, the more ROS it generates (Bejarano et al., 2011). In addition, melatonin promotes ROS production via calmodulin; ROS production is increased when melatonin interacts with calmodulin, and chlorpromazine interrupts ROS production by interrupting the binding of melatonin to calmodulin (Radogna et al., 2009).

## 3 The anticancer molecular mechanism of melatonin

Extensive research has demonstrated the crucial involvement of melatonin in regulating neoplastic progression. Especially for people who work at night or have low melatonin secretion, the cancer incidence rate increases significantly, implying an inevitable connection between melatonin and various tumors. Second, due to the antioxidant and free radical scavenging activities of melatonin, it has good anticancer activities (Table 1).

**TABLE 1:** The anticancer activity of melatonin against different types of cancer.

Cancer type	Cell Type (Human)	MLT concentration	Mechanism of action	MLT therapeutic effect	Reference
Breast Cancer	4T1, 891	100 nM	Melatonin regulates breast cancer progression through the lnc010561/miR-30/FKBP3 axis.	Inhibition of cell cycle	Liu et al. (2020a)
	MDA-MB-231	5 mM	Melatonin can induce autophagy in MDA-MB-23 breast cancer cells.	Suppress Proliferation	Wu et al. (2022)
	HCC1954(PIK3CA, H1047R), MDA-MB-453(PIK3CA, E545K), MDA-MB-361(PIK3CA, E545K), MCF7(PIK3CA, E545K)	4 mM	Melatonin enhances the cytotoxic effects of lapatinib by promoting the unfolded protein response (UPR) induced by excessive EnR stress and excessive accumulation of ROS.	Suppress Proliferation	Sang et al. (2021)
Ovarian Cancer	OVCAR3	4.8 mM	Melatonin inhibits the PI3K/Akt signaling pathway and exacerbates oxidative stress to increase apoptosis in OVCAR-3 cells.	Promote Apoptosis	Baghal-Sadriforoush et al. (2022)
	SK-OV-3, HO-8910pm	100 μm	Melatonin inhibition of the NE/AKT/β-catenin/SLUG axis reduced abdominal tumor burden in ovarian cancer.	Suppress Proliferation	Bu et al. (2020)
	SKOV-3	4 mM	Melatonin induces cell cycle arrest by reducing DNA content in S and G2/M phases in SKOV-3 cells.	Inhibition of Metastasis	Cucielo et al. (2023)
	CaKi-1, ACHN, U87MG, HCT116, PC3	4.8 mM	Melatonin upregulates ovarian tumor domain protein 1 (OTUD1) to stabilize pro-apoptotic Bcl-2 proteins and induce cell apoptosis.	Promote Apoptosis	Woo et al. (2022)
Lung Cancer	H1299, A549, H460, BEAS-2B	100 μm	Melatonin reduces the expression of circ_0017109 by directly activating miR-135b-3p to downregulate TOX3 expression and inhibit the proliferation of non-small cell lung cancer cells.	Suppress Proliferation	Wang et al. (2022b)
	H23, A549	250 μm	Melatonin and its derivative ACT reduced the expression of dry proteins Oct-4, Nanog, and β-catenin by decreasing the phosphorylation of AKT.	Inhibition of Metastasis	Phiboonchaiyanan et al. (2021)
	A549, PC9, LLC1	1 mM	Melatonin enhances mitochondrial energy metabolism by stimulating sirtuin 3 (Sirt3) to increase acetone and pyruvate dehydrogenase complex PDH activity, thereby significantly reversing the Warburg effect.	Promote Apoptosis	Chen et al. (2021a)
	A549	1 nM	Melatonin inhibits irradiation-induced apoptosis in A549 cell line.	Promote Apoptosis	Kahkesh et al. (2020)
	A459, CL1-5	3 mM	Melatonin downregulates EMT by suppressing the expression of Twist/Twist1 (Twist family bHLH transcription factor 1).	Inhibition of Metastasis	Chao et al. (2019)
Bladder Cancer	T24, RT4, HT1197, HT1376	1 mM	Melatonin inhibits bladder cancer cell migration and invasion by downregulating ZNF746-regulated MMP-9/MMP-2 signaling.	Inhibition of Metastasis	Chen et al. (2019)
	T24, 5637, UM-UC3	4 mM	Melatonin inhibits the glycolytic enzyme ENO1 and suppresses bladder cancer.	Suppress Proliferation	Shen et al. (2023)
	T24, UM-UC-3	100 μm	Melatonin inhibits cell prion protein (PrP) and suppresses bladder cancer.	Suppress Proliferation	Yang et al. (2023)
Squamous cell carcinoma of the head and neck	Cal-27, SCC9	100 μm	Melatonin drives apoptosis by increasing mitochondrial ROS generated through reverse electron transport.	Promote Apoptosis	Florido et al. (2022a)
	Cal-27, SCC9	1500 μm	Melatonin increases oxidative phosphorylation (OXPHOS) and inhibits glycolysis in HNSCC, leading to increased ROS production, apoptosis, and mitochondrial autophagy.	Promote Apoptosis	Guerra-Librero et al. (2021)
	HN6, HN12, HN30	5 mM	High-dose melatonin blocks FGF19/FGFR4 signaling	Suppress Proliferation	Lang et al. (2021)
	SCC-15	2 mM	Melatonin inhibits OSCC invasion and migration by blocking fibroblast growth factor 19 (FGF19)	Inhibition of Metastasis	Wang et al. (2021)
	SCC-15	2 mM	Melatonin induces apoptosis and ferroptosis by increasing the levels of LC3A/B, cleaved caspase-3, and PARP1 proteins.	Promote Apoptosis	Wang et al. (2023b)
	SCC-25	4 mM	Melatonin increased the levels of autophagy markers such as LC-3B and Beclin-1, inducing cell apoptosis.	Promote Apoptosis	Sung et al. (2020)
	THP-1, SCC-15	2 mM	Melatonin inhibits the development of oral squamous cell carcinoma by interrupting the MIF/NLRP3/IL-1β signaling pathway promoted by macrophages.	Inhibition of Metastasis	Wang et al. (2023d)
	SCC-9, HOK	1 mM	Melatonin induces miR-25-5p expression by directly targeting developmental downregulation protein 9 (NEDD9) expressed in neural progenitor cells.	Suppress Proliferation	Wang et al. (2020)
Gastric Cancer	AGS	4 mM	Melatonin induces apoptosis by upregulating the PERK/eIF2α pathway and downregulating the NF-κB pathway.	Promote Apoptosis	Li et al. (2022a)
prostate cancer	LNCaP, C4-2, 22RV1, PC3, DU145	1 mM	Melatonin significantly reduced the expression of carboxyesterase 1 (CES1), thereby reducing lipid droplet (LD) accumulation. This was achieved by increasing endoplasmic reticulum stress, reducing androgen synthesis, and promoting cell apoptosis.	Promote Apoptosis	Zhou et al. (2021)

### 3.1 Inhibition of tumor cell proliferation and cycle arrest

Cell proliferation refers to an increase in the number of cells. During tumor growth, abnormal cell proliferation capacity is significantly enhanced. The rapid expansion of cancer cells indicates that the disease is more invasive and spreads faster. Changes in the expression or activity of cell cycle-related proteins are the main markers of proliferation (Jarrett et al., 2018). Numerous studies have shown that MLT can inhibit cell proliferation-related pathways (CDK5 glycosylation, P21, P53, Smad3, etc.) and suppress the cell cycle (G2/M), thereby hindering cell proliferation. For example, Melatonin exerts anti-neoplastic effects in bladder carcinoma through selective suppression of O-GlcNAc post-translational modification on cell cycle-dependent kinase 5 (CDK5), thereby disrupting malignant cell cycle progression (Wu et al., 2021). melatonin demonstrates therapeutic efficacy in gastric malignancies through coordinated downregulation of CDK2/4 oncogenic drivers (Chatterjee et al., 2024). Melatonin inhibits proliferation in the G2/M phase of the hepatocellular carcinoma cell cycle and induces apoptosis by upregulating p21 and p53 (Ammar et al., 2022). Melatonin exerts antitumor efficacy in gastric malignancies through selective downregulation of Smad3-mediated proliferative signaling, effectively disrupting cell cycle progression in neoplastic epithelia (Zhu et al., 2018). In melanoma, melatonin inhibits cell proliferation by interfering with cytoskeleton formation (Alvarez-Artime et al., 2020). Melatonin inhibits proliferation of prostate cancer cells by inhibiting SENP1 protein (Nyamsambuu et al., 2022; Ha et al., 2022). Melatonin demonstrates potent anti-neoplastic activity in cervical carcinoma through dual suppression of NF-κB-mediated inflammatory signaling and COX-2 enzymatic hyperactivity, effectively arresting malignant epithelial proliferation (Minocha et al., 2022). In breast cancer, melatonin promotes breast cancer cell apoptosis through downregulation of Delta-like ligand 4 (Rajabi et al., 2020). Melatonin inhibits gastric cancer proliferation by inhibiting estrogen receptor 1 (ESR1) in bisphenol S-induced gastric cancer production (Wang Y. et al., 2023). In endometrial cancer, melatonin inhibits endometrial cancer proliferation by upregulating GATA-binding protein 2 (Liao et al., 2024). In a mouse model of pancreatic cancer, melatonin supplementation inhibited tumor growth by up to 65%, while blocking endogenous melatonin accelerated tumor growth (Chan et al., 2023).

### 3.2 Induction of apoptosis and autophagy

In the development and progression of cancer,the anti-apoptosis ability of tumorigenesis is significantly enhanced. Melatonin usually promotes apoptosis by regulating apoptosis-related proteins (caspase family, bax, bcl-2, C-myc, etc.) and inducing endoplasmic reticulum stress. Melatonin orchestrates dual antitumor mechanisms in thyroid carcinoma by simultaneously inducing programmed cell death pathways and suppressing mitogenic signaling cascades, effectively disrupting neoplastic homeostasis (Shih et al., 2021). Melatonin orchestrates dual antitumor mechanisms in cervical carcinoma by initiating endoplasmic reticulum stress-mediated unfolded protein response (UPR) while concurrently activating caspase-dependent apoptotic pathways in neoplastic epithelia (Song and Wang, 2023). ROS trigger apoptosis by activating the pro-apoptotic proteins caspase-3/7/9 and cleaved PARP, disrupting mitochondrial function, and in pancreatic cancer melatonin-induced ROS enhance apoptosis through the mitochondrial pathway (Florido et al., 2022b).

Induction of tumor cell autophagy is a new possibility for studying the potential therapeutic mechanisms of tumors (Liu et al., 2023). Autophagy is a type of programmed cell death and a relatively conserved catabolic process within cells. In cancer, autophagy maintains genomic stability, suppresses the accumulation of oncogenic proteins, and prevents tumorigenesis (Li et al., 2020). Melatonin promotes autophagy in cancer cells by regulating key proteins of autophagy (Beclin-1, LC3-II, ATG7, etc.). During autophagic flux, cytoplasmic LC3-I undergoes lipid conjugation via the ubiquitin-like system (Atg7/Atg3 cascade) to form autophagosome membrane-bound LC3-II. This stoichiometric conversion serves as a quantifiable biomarker for autophagosome biogenesis monitoring, establishing LC3 lipidation as the gold-standard metric in autophagy assessment. Melatonin can induce autophagy in OSCC cells alone or in concert with other drugs (Sung et al., 2020; Wang et al., 2023b), Melatonin membrane receptor induces increased autophagy of TFE3 and induces apoptosis in tongue squamous cell carcinoma (TSCC) (Fan et al., 2018). In gastric cancer, melatonin promotes apoptosis in gastric cancer cells by upregulating HSF1 protein (Li W. et al., 2022). melatonin is also involved in the autophagy process of breast cancer cells, D Wu et al. found that significantly increased anti-apoptotic proteins, LC3- ΙΙ/LC3-Ι ratio of autophagy marker LC3, and the expression of Beclin1 when it was used in the induction of autophagy in breast cancer cells through the combination of MLT and autophagy inhibitor 3-MA. decreased, indicating that autophagy inhibitors can reverse the inhibitory effect of melatonin on breast cancer and that melatonin inhibits breast cancer by inducing autophagy (Wu et al., 2022). Combined treatment with melatonin and Andrographis paniculata in rectal cancer results in an increased LC3-II to LC3-I ratio and promotes autophagy and apoptosis in rectal cancer cells (Zhao et al., 2022).

### 3.3 Inhibition of metastasis and anti-angiogenesis

The acquisition of epithelial-mesenchymal transition (EMT) characteristics represents a critical indicator of metastatic potential in malignant neoplasms. Among the regulatory network governing this process, Twist and Snail transcription factors have been identified as pivotal molecular regulators that orchestrate both EMT activation and the dissemination of cancerous cells (Gundamaraju et al., 2022),and have been shown to target Twist to inhibit the EMT of lung cancer cells, thereby effectively controlling lung cancer metastasis (Chao et al., 2019). In a seminal study conducted by Karadas et al. Their experimental findings revealed that MLT administration effectively suppressed hepatic and pulmonary metastatic dissemination in murine models of mammary carcinoma. Furthermore, the investigation demonstrated that this indoleamine compound exerted dual inhibitory effects on both angiogenesis and neoplastic proliferation in breast cancer specimens (Karadas et al., 2021). Endothelin-1 (ET-1) inhibits osteoblast differentiation, melatonin inhibits prostate cancer bone metastasis by inhibiting ET-1, making melatonin a promising therapy (Lin et al., 2024). The ECM is a specialized extracellular matrix in the basement membrane that surrounds solid tumors and serves as a structural barrier that anatomically separates the tumor from the surrounding normal tissue (Mond et al., 2020). Matrix metalloproteinases (MMPs) play an important role in explaining the ECM, and melatonin inhibits BC development by downregulating the AKT/MMP9 signaling pathway (Chen et al., 2019). Oncogenic transcription factor (FOSL1) can regulate EMT in a variety of tumors (Sobolev et al., 2022),synergizes with PDL-1 in HNSCC, and significantly inhibits EMT through the ERK1/2/FOSL1 pathway (Luo et al., 2022). In breast cancer, melatonin treatment reduces STAT3 phosphorylation, thereby inhibiting epithelial mesenchymal transformation and metastasis (Das et al., 2024). Melatonin inhibits chondrosarcoma cell proliferation by inhibiting matrix metalloproteinase 7 (MMP7) (Nguyen et al., 2023).

Increased angiogenesis is an important factor in promoting tumorigenesis. Tumor development is usually inhibited by direct or indirect inhibition of angiogenic factors (VEGF, PDGF, HIF1, etc.), and melatonin can achieve this function. Melatonin inhibits the progression of hepatocellular carcinoma in rats by decreasing VEGF levels (Bahaa Eldeen et al., 2023). Melatonin inhibits tumorigenesis by reducing HIF1 levels (Kim et al., 2013; Park et al., 2010). Melatonin enhances the inhibitory effect of netazumab on glioblastoma by inhibiting EGFR, Melatonin significantly reduces microvascular density and vascular endothelial growth factor (VEGF) expression levels in mouse tumors, inhibiting tumor angiogenesis (Wang et al., 2024).

### 3.4 Metabolic reprogramming

**FIGURE 2:**
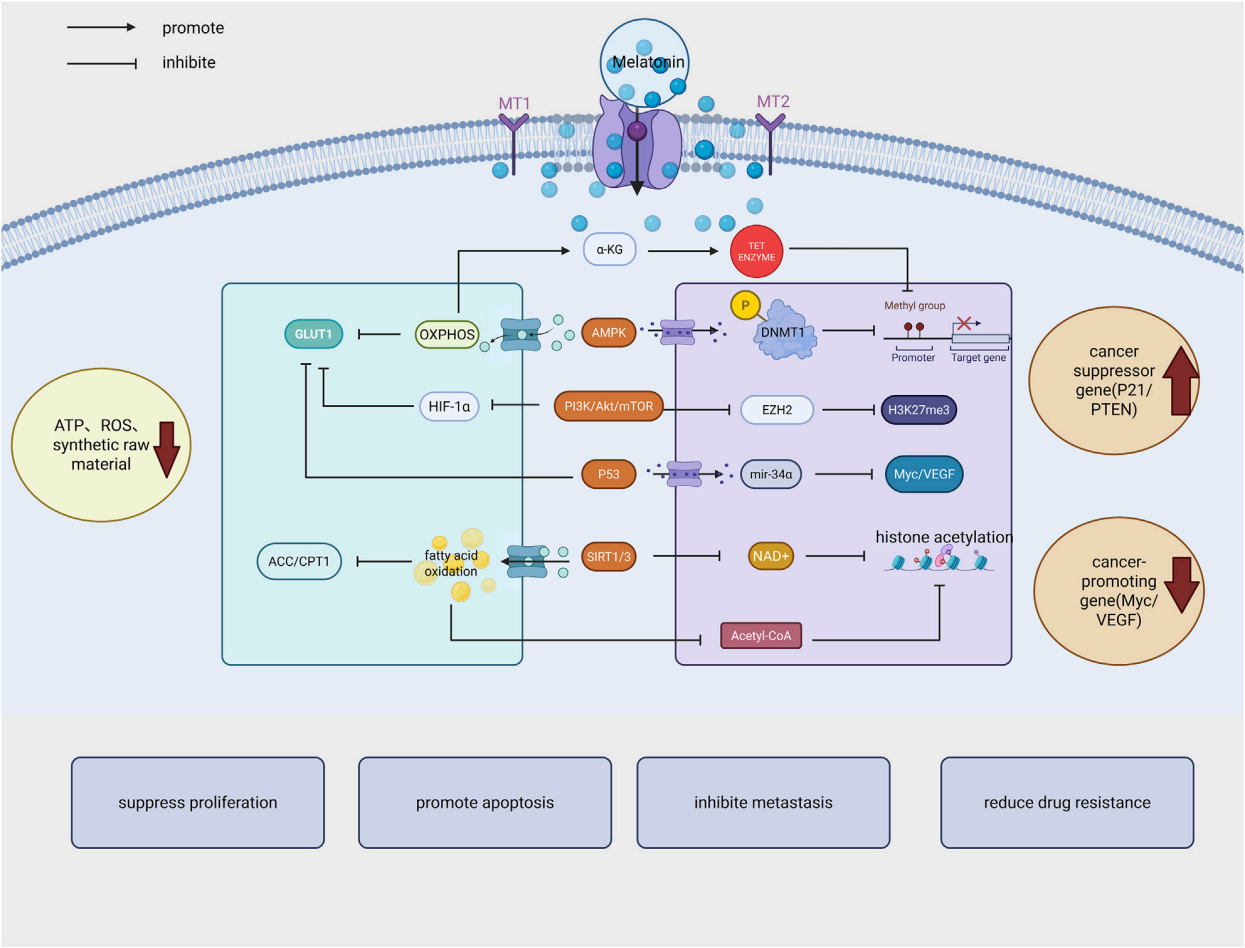
Melatonin exerts its anticancer effects by inhibiting cancer cell proliferation, promoting apoptosis, reducing metastasis and drug resistance through multiple mechanisms, including regulation of energy metabolism, epigenetic modification, signaling pathways, and tumor suppressor gene expression. (GLUT1: Glucose transporter 1; OXPHOS:oxidative phosphorylation; AMPK: AMP-activated protein kinase; HIF-1α: Hypoxia-inducible factor 1α; SIRT1/NAD+: Deacetylase; EZH2 (H3K27me3): Histone methyltransferase; DNMT1: DNA methyltransferase 1; ACC/CPT1: Acetyl-CoA carboxylase and carnitine palmitoyltransferase 1). Created in BioRender. cao, y. (2025) https://BioRender.com/bqm60d5.

Tumor metabolic reprogramming is the metabolic reprogramming of cancer cells to adapt to hypoxia and nutrient deficiencies, Melatonin has the ability to regulate cancer cell metabolic reprogramming (see Figure 2), in which mitochondrial metabolism is one of the most important factors in cancer development and inhibits the development of HNSCC by regulating mitochondrial metabolism and function (Guerra-Librero et al., 2021). MLT can be combined with vitexoporfin to regulate mitochondrial function and inhibit the growth and stemness of HNSCC (Shin et al., 2022).

In tumor cells, aerobic glycolysis (Warburg effect) leads to tumor promotion through glucose uptake and lactate production for a rapid tumor energy supply. Through its modulatory effects on energy metabolism pathways, melatonin suppresses key oncogenic factors implicated in ovarian carcinoma progression and metastatic potential. This is achieved by downregulating both aerobic glycolytic processes and glutamine degradation mechanisms, thereby fundamentally reshaping the metabolic profile of malignant ovarian cells,In a nude mouse tumor suppression model of ovarian cancer cells, the tumor volume in the melatonin treatment group was reduced by 50% (Silveira et al., 2024). ENO1, best known for catalyzing glycolysis’ ninth enzymatic step, has been identified as a melatonin-regulated protein that mediates downstream metabolic processes, which is mainly involved in the glycolytic process of tumor cells to provide energy support for the survival of tumor cells (Huang et al., 2022). Melatonin induces toxicity of the chemotherapeutic drug gemcitabine in BC cells by silencing the ENO1 upstream factor PPARγ,In a bladder cancer xenograft model, the tumor growth inhibition rate in the melatonin treatment group reached 58%,providing a new perspective for MLT treatment of BC (Shen et al., 2023). Melatonin inhibits smooth muscle sarcoma by suppressing aerobic glycolysis, inhibiting the uptake of linoleic acid (LA) and the release of 13-hydroxy octadecadienoic acid (13-HODE), thereby inhibiting its proliferation and invasion (Mao et al., 2016).

Additionally, the Warburton effect appears to interact with mitochondrial oxidative reactions. Some researchers have speculated that melatonin may act as a glycolytic agent similar to the anticancer drug DCA, targeting the mitochondria of metabolically reprogrammed cancer cells. Melatonin upregulates the pyruvate dehydrogenase complex (PDC), reprogramming pyruvate in mitochondria, promoting the metabolism of pyruvate to acetyl-CoA in mitochondria, and inhibiting the Warburg effect (Reiter et al., 2020).

Fat metabolism is an important part of cancer metabolic reprogramming. Under hypoxic conditions, the rate of fatty acid synthesis increases in cancer cells (Xu et al., 2023). Carboxylesterase 1 (CES1) is an enzyme that inhibits fat accumulation, induces lipid metabolism and increases endoplasmic reticulum stress (Gan et al., 2023),Melatonin can target PCa by upregulating the expression of CES1 to achieve this function (Zhou et al., 2021).

Folic acid drives tumor development by increasing nucleotide synthesis and methylation capacity. In tumor metabolic reprogramming, nucleotide metabolism is linked to glucose metabolism and amino acid metabolism. Methylenetetrahydrofolate dehydrogenase 1-like (MTHFD1L) is a metabolic enzyme that regulates the folate cycle from format production (Agarwal et al., 2019),It is a downstream target of MLT, which was found by Cui et al. to inhibit the development of HNSCC by inhibiting the expression of MTHFD1L mainly through downregulation of CREB1 phosphorylation,In a head and neck squamous cell carcinoma xenograft model, melatonin inhibited tumor growth by more than 60% (Cui et al., 2021).

## 4 Epigenetic regulation

Epigenetic modifications are primarily categorized into four types: DNA methylation, histone modifications, chromatin remodeling, and non-coding RNA-induced modifications (Xu et al., 2023). DNA methylation maintains dynamic equilibrium within the body to ensure normal physiological functions. In tumor cells, abnormal methylation can lead to the activation of certain proto-oncogenes and the silencing of tumor suppressor genes. The key regulatory enzymes of DNA methylation are DNA methyltransferases (DNMTs), and the key enzymes for active DNA methylation are TET enzymes. In various cancers, the balance between DNA methylation and demethylation is disrupted, leading to impaired expression of DNMT and TET. Melatonin regulates the activity of DNMT and TET, thereby influencing the expression of tumor suppressor genes and oncogenes. Melatonin promotes the expression of DNMT1 and epigenetic suppression of the transcription of the tumor suppressor gene ARHI (Ras homolog 1), thereby reducing the sensitivity of breast cancer to paclitaxel chemotherapy (Xiang et al., 2019). Melatonin reduces the expression of transport proteins and the resistance of brain tumor stem cells to chemotherapy drugs by inducing methylation of the promoter of ABCG2/BCRP, a member of the adenosine triphosphate-binding box (ABC) superfamily (Martín et al., 2013).

Histone modifications influence chromatin structure and gene transcription. The N-terminal regions of histones can undergo post-translational modifications such as methylation, acetylation, lactylation, glycosylation, propionylation, or butyrylation, which alter gene expression. Melatonin exerts its anticancer effects by regulating histone deacetylases (HDACs) and histone acetyltransferases (HATs). Melatonin inhibits the growth of esophageal squamous cell carcinoma by suppressing histone deacetylase 7 (HDAC7) (Ma et al., 2022). HDAC9 knockdown further enhanced the anticancer activity of melatonin treatment in non-small cell lung cancer (Ma et al., 2019). Melatonin inhibits the growth of glioblastoma stem cells by suppressing the NOTCH1 signaling axis induced by histone methyltransferase EZH2 (Zheng et al., 2017). Glycosylation is a post-translationally modified form of the metabolic flux of glucose or other monosaccharides (Pinho and Reis, 2015). Dysregulation of glycosylation triggers tumor development, and O-GlcNAcylation is usually a biomarker of dysregulated glycosylation (Chatham et al., 2021). MLT significantly downregulates O-GlcNAcylation, a dysregulated glycosylation marker, to reduce BC cell proliferation and pro-apoptosis (Wu et al., 2021).

Non-coding RNAs (ncRNAs), consisting of microRNAs (miRNAs), long non-coding RNAs (lncRNAs), and circular RNAs (circRNAs), have been increasingly recognized as crucial for various biological processes in recent years. Long non-coding RNAs (lncRNAs) represent a class of epigenetically active molecules that orchestrate post-transcriptional gene regulation through competitive sequestration of chromatin modifiers and microRNAs. This RNA-protein interaction paradigm positions lncRNAs as promising therapeutic candidates for targeted oncogenic pathway modulation in precision oncology (McCabe and Rasmussen, 2021). Melatonin coordinates lncRNA to inhibit breast cancer development, and FK506-binding protein (FKBP3) and lnc010561 act as competing endogenous RNAs (ceRNAs) for the tumor suppressor mir-30, which regulates breast cancer development because of the significant downregulation of FKBP3 by melatonin (Liu P. et al., 2020). Melatonin suppresses triple-negative breast cancer (TNBC) oncogenesis through competitive ceRNA-mediated modulation of the lnc049808/miR-101/FUNDC1 mitophagic signaling axis, effectively disrupting mitochondrial homeostasis in malignant epithelia (Yang et al., 2021).

Cyclic RNA is highly conserved and very stable; therefore, it is considered a promising tumor biomarker for precision medicine. Hsa_circ_0017109 Increased expression is a biological process that promotes hyperproliferation and metastatic invasion of lung carcinoma. Wang et al. found that downregulation of Hsa_circ_0017109 expression can effectively inhibit the development of lung cancer, and melatonin plays an exact role (Wang Y. et al., 2022).

Melatonin inhibits cancer cell proliferation to promote apoptosis by up-regulating pro-apoptosis-related miRNAs and down-regulating anti-apoptosis miRNAs. Melatonin also impedes tumor progression through miRNA regulation of pathways related to cancer progression. In addition, melatonin inhibits GC development by suppressing the exosome miR-27b-3p (Zhang et al., 2023). Melatonin inhibits malignant progression of glioblastoma by negatively regulating its downstream target PIM1 through upregulation of mir-16-5p (Yan et al., 2022). Melatonin inhibits human glioblastoma development by regulating HIF1-α/VEGF/MMP9 signaling through the regulation of differentially expressed vascular miRNAs in 6 (Doğanlar et al., 2021). Figure 2 summarizes the interaction mechanisms between melatonin and metabolic reprogramming and epigenetic regulation.

## 5 Tumor immune microenvironment

The tumor immune microenvironment (TME) is an integral part of cancer progression, influencing metastasis and treatment response. It consists of multiple cell types, extracellular matrix components, and signaling molecules that interact to promote cancer cell growth, invasion, metastasis, and treatment resistance (Bilotta et al., 2022; Jin and Jin, 2020).

Immunosuppressive regulatory T cells (Tregs) are a major mechanism of tumor immune escape (Qin et al., 2024). Targeting Tregs plays an important role in tumor immune escape and has significant antitumor effects. IL-10 and TGF-β are two key cytokines released by Tregs (Sawant et al., 2019). Melatonin reverses immune suppression by reducing the secretion of TGF-β by tumor cells and decreasing the accumulation of myeloid-derived suppressor cells (MDSCs). Melatonin acts on the interactions between Tregs and other cells, thereby eliminating Treg function. Melatonin has also been found to induce the release of inflammatory cytokines such as IFN-γ and TNF-α, which not only promote the proliferation of CD8^+^ T lymphocytes but also inhibit the proliferation of Tregs (Mu and Najafi, 2021).

Macrophages are divided into two types: classically activated M1 macrophages and selectively activated M2 macrophages (Pan et al., 2020). M1-type macrophages primarily release pro-inflammatory factors, while M2-type macrophages produce anti-inflammatory cytokines such as IL-4, IL-10, and IL-13 within tumors (Murray et al., 2014). Tumor-infiltrating macrophages (TAMs) are the main macrophages in tumors and exhibit M2-type characteristics (Fu et al., 2020). Melatonin can inhibit the release of cytokines such as IL-6, IL-10, and IL-12 by macrophages. After melatonin treatment, the inhibition of the TLR9/ERK1/2 pathway in macrophages plays a key role in preventing the release of pro-inflammatory cytokines (Xu et al., 2018). In addition, melatonin can also inhibit the expression of other inflammatory mediators by macrophages. MLT treatment increased the secretion of TNF-α and CXCL10 by macrophages, thereby inhibiting the growth of gastric cancer cells (Wang K. et al., 2023).

T lymphocytes include various types of cells, such as CD4^+^ T lymphocytes (i.e., type 1 and type 2 helper T cells), Th17 cells, and cytotoxic CD8^+^ cells. Th1 cells release inflammatory cytokines, such as IFN-γ, TNF-α, and IL-2. These cytokines activate the immune response of NK cells and CD8^+^ T lymphocytes and promote the proliferation of CD8^+^ T lymphocytes. In contrast to Th1 cells, Th2 cells release anti-inflammatory cytokines such as IL-4 and IL-10 (Zhang et al., 2014). In addition, MLT treatment of gastric cancer cells leads to the production of exosomes, which promote the recruitment of CD8^+^ T cells to the tumor site, thereby inhibiting tumor growth (Wang K. et al., 2023). Melatonin therapy significantly increased the number of CD3^+^ CD4^+^ and CD3^+^ CD8^+^ T cells, but reduced the infiltration of Ly6G + F4/80- myeloid-derived suppressor cells (MDSCs), significantly inhibiting the growth of non-small cell lung cancer (Chao et al., 2021).

Natural killer (NK) cells are key immune cells in the fight against cancer cells. NK cells kill cancer cells by releasing inflammatory cytokines such as IFN-γ and TNF-α (Vallentin et al., 2015). NK cells may be influenced by molecules released in the tumor microenvironment, thereby promoting angiogenesis and tumor growth (Zhang et al., 2020). Melatonin or its agonists, such as agomelatine and remimegrotin, can promote the release of IL-2, which is a key stimulatory factor for NK cell proliferation (Srinivasan et al., 2011).

**FIGURE 3:**
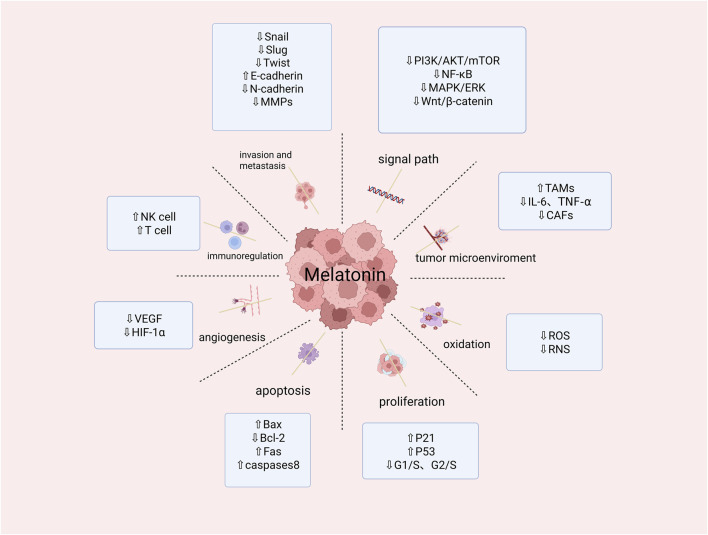
Summary of melatonin activity in restraining cancer hallmarks. (Snail, slug,twist,NF-κB:transcription factor; E-Cadherin,Bax:pro-apoptotic protein; N-Cadherin:inhibitor of apoptoasis protein; MMPs:matrix metalloproteinases; PI3K/AKT/mTOR:phosphatidylinositol-3-kinase/Protenin KinaseB/Mammalian Target of Rapamycin signal channel; MAPK/ERK:signal channel; Wnt/β-catenin:signal channel; TAMs:Tumor-Associated Macrophages; IL-6,TNF-α:proinflammatory cytokine; CAFs:Cancer-Associated Fibroblasts; ROS:Reactive Oxygen Species; RNS:Reactive Nitrogen; P21,P53:cancer suppressor genen; G1/S,G2/S:cell cycle; Bcl-2:anti-apoptosis gene; Fas:tumor necrosis factor; Caspase 8:cysteine protease; VEGF:vascular endothelial growth factor; HIF-1α:the transcription factor hypoxia-inducible factor 1α; NK cell:identify tumor cells; T cell:kill tumor cells). Created in BioRender. cao, y. (2025) https://BioRender.com/u29o844.

Cancer-associated fibroblasts (CAFs) regulate immune responses, alter the composition of the extracellular matrix, and promote angiogenesis to drive tumor progression and metastasis (Chen D. et al., 2021). CAFs can promote endothelial cell proliferation by directly secreting vascular endothelial growth factor (VEGF) and fibroblast growth factor (FGF) through exosomes. CAFs also secrete chemokine matrix cell-derived factor 1 (SDF-1), which recruits endothelial progenitor cells (EPCs) into peripheral blood and guides their migration to the tumor periphery. Melatonin inhibits the infiltration of triple-negative breast cancer-associated fibroblasts (CAFs) by downregulating the expression of laminin beta-3 (LAMB3) and the C-X-C chemokine ligand 2 (CXCL2) (Lai et al., 2024). IL-8 is primarily expressed in CAFs. Melatonin inhibits IL-8 expression in CAFs by suppressing the NF-κB pathway and AKT pathway, thereby directly or indirectly inhibiting tumor progression (Liao et al., 2023). Figure 3 summarize the role of melatonin in cancer hallmarks.

## 6 MLT and signaling pathways

**FIGURE 4:**
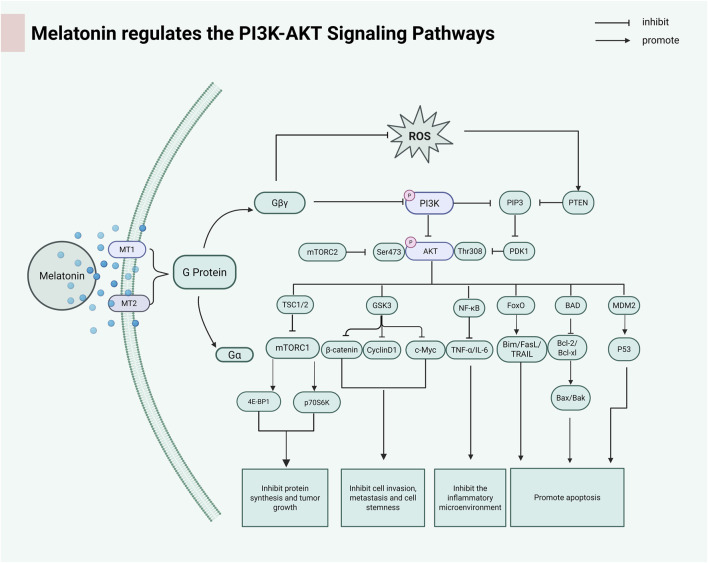
Melatonin activates G proteins by binding to the MT1 and MT2 receptors. The dissociation of the Gβγ subunit further activates the downstream PI3K-AKT signaling pathway. AKT regulates the mTOR complex through phosphorylation, thereby affecting cell growth, metabolism, and survival. (PIP3:Phosphatidylinositol (3,4,5)-trisphosphate; PTEN:anticancer protein; PDK1:3-Phosphoinositide-Dependent Protein Kinase 1; TSC1/2:Tuberous Sclerosis Complex; GSK3:Glycogen Synthase Kinase 3; FoxO:Forkhead box class O; BAD:Pro-apoptotic BCL-2 family; MDM2:p53 tumor suppressor protein; 4EBP1:Translation suppression protein; p70s6k:Serine/threonine protein kinase). Created in BioRender. cao, y. (2025) https://BioRender.com/x11d1oe.

The PI3K/AKT/mTOR (PAM) signaling axis functions as an evolutionarily conserved regulatory network coordinating pro-survival mechanisms, mitogenic processes, and cell cycle regulation through integrated phosphorylation cascades (Glaviano et al., 2023). Through its regulatory effects on the PI3K/AKT signaling cascade, melatonin enhances programmed cell death in ovarian carcinoma cells, thereby suppressing tumor progression and malignant transformation (Baghal-Sadriforoush et al., 2022). MLT inhibits AKT pathway activation by decreasing MMD2, a downstream target of AKT. Inhibition of mTOR induced autophagy in cancer cells through activation of ULK1, leading to Beclin-1 phosphorylation (Pourbarkhordar et al., 2024). MLT plays a major role in inhibiting bladder cancer growth, proliferation and invasion/metastasis by inhibiting Notch/JAG2 signaling through upregulation of PI3K/AKT/mTOR downstream signaling (Chen et al., 2020). Melatonin activates the PI3K/AKT axis, leading to upregulation of ETS and inhibition of apoptosis in hyperoxia-exposed lung cancer cells (He et al., 2023). Figure 4 summarizes the regulatory mechanism of melatonin on the PI3K/AKT signaling pathway.

Abnormal activation of Wnt/β-catenin signal transduction is closely related to the occurrence and development of cancer (Yu et al., 2021). Melatonin (MLT) paradoxically enhances metastatic progression in ovarian carcinoma through NE/AKT/β-catenin/SLUG axis potentiation, yet concurrently attenuates chemotherapy-related sequelae (CRS)-driven oncogenesis via SLUG-mediated epithelial-mesenchymal transition (EMT) suppression in preclinical models (Bu et al., 2020). MLT combined with Andrographis paniculata in the treatment of colon cancer, the main mechanism is to induce cell death by inhibiting β-catenin expression and its downregulated signals Cyclin D1 and c-Myc (Sokolov et al., 2022).

Melatonin inhibits cervical cancer cell proliferation by suppressing NF-κB pro-inflammatory transcription factor expression. Melatonin demonstrates oncostatic efficacy in hepatocellular carcinoma (HCC) through dual-pathway modulation: suppressing NF-κB transcriptional activation while attenuating TNF-α-mediated proinflammatory cascades (Ozturk et al., 2023).

## 7 Synergize with chemotherapeutic agents

In order to further study the synergistic mechanism of melatonin and chemotherapeutic agents, optimize the combined treatment regimen, improve therapeutic efficacy, and reduce side effects, a large number of studies have been conducted. Cisplatin, a platinum-based chemotherapeutic agent, acts on tumorigenesis mainly by inducing DNA damage and apoptosis, to which tumors are prone to develop resistance and its main side effect is that it affects the secretion function of oral salivary glands, resulting in a series of oral-associated diseases (Dasari and Tchounwou, 2014). MLT has superior anti-inflammatory and antioxidant effects, and the combination of melatonin and cisplatin treatment significantly attenuates the destruction of the submandibular gland due to the chemotherapy of cisplatin and reduces the side effects (Badawy et al., 2024). In addition, melatonin enhanced the sensitivity and efficacy of cisplatin for osteosarcoma chemotherapy (Hosseini et al., 2022). MLT attenuated acute kidney injury induced by cisplatin chemotherapy (Kim et al., 2019). Injury to renal tubular epithelial cells is also frequently seen in cisplatin treatment; fatty acid oxidation (FAO) supplies energy to renal tubular epithelial cells, where peroxisome proliferator receptor alpha (PPARα) is a major regulator of FAO (Robbins and Nie, 2012),Melatonin increased PPARα gene and FAO expression and reduced cisplatin-generated acute kidney injury (Li N. et al., 2022).

Melatonin reduces the toxicity of chemotherapeutic drugs while at the same time is significantly anti-decaying and has become a new means of adjuvant chemotherapy for the elderly (Ma et al., 2020). 5-Fluorouracil (5-FU) has become one of the most commonly used chemotherapeutic drugs for cancer treatment, and the use of melatonin in combination with 5-FU reduces the toxicity of the drug and decreases drug resistance (Mafi et al., 2023). Lapatinib is commonly used in the treatment of HER2 positive breast cancer but is prone to recurrence due to drug resistance (Yuan et al., 2023; Yang et al., 2022; Zhang et al., 2024).

Paclitaxel (PTX) is a classic microtubule stabilizer chemotherapy drug that blocks cell mitosis, induces cancer cell apoptosis, and inhibits tumor metastasis (Abbaspour et al., 2025). However, paclitaxel has neurotoxicity and bone marrow suppression issues. In breast cancer, exposure to dim nighttime lighting (dLAN) disrupts the circadian rhythm of melatonin, which drives intrinsic resistance to paclitaxel through epigenetic mechanisms, increases STAT3 expression, and enhances breast tumors’ sensitivity to paclitaxel, inhibiting its growth (Xiang et al., 2019). Melatonin inhibits dryness by activating MT1 to suppress c-Myc, nestin, and histone methylation, thereby promoting the anticancer effect of paclitaxel in brain cancer stem cells (Lee et al., 2018). Table 1 summarizes the anticancer effects of melatonin on different types of cancer and their mechanisms of action.

## 8 Clinical trial

Melatonin has been used in anticancer clinical trials in various types of tumors, and confirmed the beneficial effects of melatonin on various types of cancer. To further promote the use of melatonin as an adjunctive therapy to traditional anticancer treatments, researchers investigated the efficacy of melatonin in clinical studies and patients (Table 2). Most clinical studies used melatonin in combination with chemotherapy or as a protective therapy, including alleviating chemotherapy-induced side effects, reducing the incidence of depressive symptoms, and improving sleep quality in cancer patients (Fatemeh et al., 2022). A prophylactic regimen of 20 mg exogenous melatonin administered 10 days prior to and during initial breast cancer adjuvant chemotherapy (ACBC) demonstrated neuroprotective efficacy, effectively counteracting treatment-induced cognitive impairment, sleep dysregulation, and depressive symptomatology (Palmer et al., 2020). Advanced cancer patients treated with MLT showed significant improvement in sleep disorders in a double-blind clinical trial (Mendis et al., 2024). Among breast cancer patients receiving chemotherapy, showed that melatonin had the ability to significantly ameliorate symptoms such as fatigue after adjuvant therapy for breast cancer (Sedighi Pashaki et al., 2023). Conversely, some clinical trials have also shown conflicting results. Cisplatin, one of the most commonly used cancer chemotherapy drugs, causes significant loss of magnesium and potassium in cancer patients. Melatonin adjunctive therapy improved the incidence of acute kidney injury and the rate of magnesium and potassium loss in urine; however, it did not demonstrate positive results in preventing acute kidney injury (Karvan et al., 2022). A clinical double-blind, phase III randomized controlled trial study indicated that melatonin adjunctive therapy can increase disease-free survival (DFS) in patients with advanced non-small cell lung cancer, but it has no significant effect on postoperative fatigue, depression, and anxiety (Seely et al., 2021). Further research is needed to explore its effectiveness. In addition, recent studies have shown that patients undergoing chemotherapy for breast cancer are prone to fatigue, and the experimental group was administered melatonin 20 mg orally from the night before the start of chemotherapy until 2 weeks after the start of chemotherapy. The results showed that melatonin did not significantly improve the patients’ symptoms of fatigue and sleep disturbance. It is thought-provoking to note that the study did not conduct serologic testing to further validate the (Mukhopadhyay et al., 2024).

**TABLE 2:** Clinical evidence of melatonin anticancer effects.

Evidence types	Research type	Conclusion	References
Positive Evidence	Randomized controlled trial	Melatonin treatment has a positive effect on sleep quality.	Fatemeh et al. (2022)
	Randomized, double-blind, placebo-controlled trial	Hormones have a neuroprotective effect on breast cancer patients undergoing chemotherapy, mitigating the adverse effects of adjuvant chemotherapy on cognitive function, sleep quality, and depressive symptoms.	Palmer et al. (2020)
	Phase III randomized clinical trial	Melatonin improves sleep in patients with advanced cancer.	Mendis et al. (2024)
	Randomized clinical trial	Melatonin reduces fatigue levels in women undergoing adjuvant therapy for breast cancer and improves quality of life.	Sedighi Pashaki et al. (2023)
	Randomized clinical trial	Melatonin prevents cisplatin-induced acute kidney toxicity.	Karvan et al. (2022)
	Randomized clinical trial	Melatonin increases the 2-year disease-free survival rate in patients with advanced lung cancer.	Seely et al. (2021)
Negative Evidence	Randomized clinical trial	Melatonin has no significant effect on 2-year disease-free survival in patients with early-stage lung cancer.	Seely et al. (2021)
	Double-blind, placebo-controlled Phase III trial	Melatonin did not prevent or significantly improve fatigue and other symptoms in patients with early breast cancer undergoing radiotherapy.	Mukhopadhyay et al. (2024)

Overall, melatonin, as an adjuvant to the main anticancer therapies, can enhance the anticancer effects and significantly improve the quality of life of cancer patients with fatigue, depression and other symptoms associated with chemotherapy. Of course, there are also some conflicting research results, which require well-designed studies with longer follow-up periods and larger sample sizes for verification.

## 9 Challenges and constraints

Absorption, metabolism, and excretion of melatonin vary from individual to individual, and secondly, the type of drug formulation needs to be considered in order to achieve clinical therapeutic benefit. Ideally, it is recommended that melatonin be administered orally at the usual bedtime time of approximately 45 min to 1 h (Arendt, 1998). Route of administration, age, hepatic function, and potential drug interactions may affect plasma melatonin levels, and melatonin sensitivity and pharmacokinetics vary from person to person; Clinical observations suggest diminished dosing ranges (0.3–0.5 mg) frequently exhibit enhanced therapeutic outcomes compared with elevated dosages across diverse patient populations (Harpsøe et al., 2015). In addition, the collection of melatonin samples in the clinic needs to vary according to the patient’s time of secretion due to differences in the timing of melatonin secretion, which greatly increases the difficulty of sample collection.

## 10 Challenges and strategies for clinical translation

### 10.1 Improvement of bioavailability

Melatonin has a short blood half-life, rapid cycling, and high hepatic metabolism. To optimize sustained therapeutic efficacy, developing controlled-release melatonin formulations with prolonged circulation half-life becomes imperative. Pharmacokinetic studies demonstrate that modified-release 2 mg oral tablets achieve peak serum concentration (Tmax) at 6 h post-administration, sustaining bioactive levels above the therapeutic threshold for 3.5 h through first-order elimination kinetics. The sublingual delivery system demonstrated accelerated melatonin absorption kinetics, achieving peak plasma concentration (Cmax) within 30 min - pharmacokinetic behavior analogous to immediate-release (IR) formulations. Comparatively, oral tablet administration exhibited reduced Cmax values but prolonged therapeutic exposure, characterized by an extended elimination half-life (t1/2) and greater area under the curve (AUC) retention (Ait Abdellah et al., 2023). Oniria, an oral extended-release form of melatonin, also significantly increased its bioavailability (Román Martinez et al., 2022).

### 10.2 Enhanced targeting

When taken orally, melatonin is rapidly metabolized by CYP450 enzymes in the liver into 6-hydroxy melatonin, with a bioavailability of only 3%–15%. Due to its high lipophilicity, melatonin distributes unevenly and tends to accumulate in adipose tissue. Additionally, its short half-life necessitates frequent dosing, which limits its clinical application (Harpsøe et al., 2015). Targeted delivery of melatonin to tumor sites using nanotechnology and nanocoupling to reduce side effects on normal tissues. Melatonin secretion decreases with age, and the use of prostate-specific membrane antigen (PSMA)-targeted nanocarriers loaded with I125 radioactive particles and encapsulated siRNAs targeting APE1 (siAPE1) and melatonin for the treatment of PCa played a good role in tumor-targeted therapy (Liu et al., 2024). Melatonin-containing lactoferrin-chitosan-etoposide nanoparticles show good efficacy in targeting colorectal cancer therapy, increasing bioavailability, and improving drug delivery (Raval et al., 2025). When encapsulated within NIR-responsive chitosan (CS) biopolymers exhibiting superior biocompatibility, melatonin triggers apoptotic cascades in gastric carcinoma through ROS-dependent PI3K/Akt/mTOR axis modulation, leveraging photothermal conversion for spatiotemporal control of therapeutic payload release (Fan et al., 2024). Polylactic acid-hydroxyacetyl copolymer (PLGA) controls degradation rate by regulating the lactic acid/hydroxyacetic acid ratio. Brown algae polysaccharide/chitosan-layered PLGA nanoparticles loaded with melatonin can induce slow release of melatonin, enhance intestinal absorption, and inhibit the progression of triple-negative breast cancer (Yen et al., 2023).

In a mouse model of prostate cancer, the tumor suppression rate of the mitochondrial-targeted nanoparticle Mito-Mel was equivalent to that of 500 mg/kg of free melatonin, representing an approximately 100-fold improvement in efficacy (Chen et al., 2025). Melatonin and gemcitabine were co-delivered in a pancreatic cancer model, achieving a tumor inhibition rate of 68%, significantly higher than the 25% observed in the free group (Ibrahim et al., 2025).

Exosomes mediate intercellular communication. Through exosomes, donor cells can transfer exogenous substances such as proteins, mRNA, microRNA (miRNA), and lipids to recipient cells (Alfonsi et al., 2018). Current research indicates that exosome-mediated drug delivery has low toxicity, low immunogenicity, and high engineering potential (Liang et al., 2021). Recent research has developed engineered M2 macrophage-derived exosomes loaded with melatonin, which can effectively target periodontal inflammation sites and mediate immune reprogramming to promote macrophage repolarization (Cui et al., 2023). Melatonin-pretreated mesenchymal stem cell-derived exosomes (MT-Exo) can suppress inflammation by increasing the ratio of M2 polarization to M1 polarization through activation of the PTEN/AKT signaling pathway, and can promote diabetic wound healing (Liu W. et al., 2020). Endothelial cell-derived primary exosomes mediate melatonin inhibition of vascular calcification and vascular aging in an m6A methylation-dependent manner (Shan et al., 2024). However, current research on the use of exosome-encapsulated melatonin for targeted delivery in cancer is minimal, and we anticipate further studies on melatonin in this area.

### 10.3 Individualized treatment

Due to the differences in the timing of patients’ melatonin secretion, patients who are sensitive to melatonin are screened for melatonin therapy through genomics, proteomics, and other techniques. The administration time and dose of melatonin are optimized according to the patient’s circadian rhythm, tumor stage, and type.

### 10.4 Development of novel melatonin analogs

Developing melatonin analogs through chemical modification to enhance their anticancer activity and stability. To design melatonin derivatives with multi-targeted effects that simultaneously act on multiple key pathways in tumors to achieve multiple anti-tumor effects.

## 11 Conclusion and perspectives

Melatonin, which is a natural hormone with multiple anticancer activities, has made significant progress in cancer prevention research in terms of its role and mechanism. MLT inhibits cancer progression through anti-inflammatory and antioxidant modulation of the immune system, induction of apoptosis, and synergistic chemotherapeutic agents. Targeting MLT is prominent and can effectively reduce side effects and improve bioavailability. Although MLT has been shown to have therapeutic effects on certain cancers in *ex vivo* and *in vivo* studies, its molecular mechanism remains unclear, and most of the studies on MLT have focused on the cellular level, with the direct target in tumors still unknown. In addition, due to the time-dependent and concentration-dependent nature of melatonin, although it has been approved by the FDA for the treatment of insomnia and other therapies, no substantial progress has been made in its clinical use in cancer. Currently, there have been related studies using nanosystems to improve the targeting and utilization of melatonin, so melatonin is also a natural anticancer hormone worthy of in-depth study in the future.
